# Turning a blind eye: the mobilization of radiology services in resource-poor regions

**DOI:** 10.1186/1744-8603-6-18

**Published:** 2010-10-14

**Authors:** Duncan Smith-Rohrberg Maru, Ryan Schwarz, Andrews Jason, Sanjay Basu, Aditya Sharma, Christopher Moore

**Affiliations:** 1 Nyaya Health, Bayalpata Hospital, Ridikot VDC, Achham, Nepal; 2 Brigham and Women's Hospital, Department of Medicine, Boston, MA, USA; 3 Children's Hospital of Boston, Department of Medicine, Boston, MA, USA; 4 Yale University School of Medicine, New Haven, CT, USA; 5 Department of Medicine, University of California San Francisco & Division of General Internal Medicine, San Francisco General Hospital; 6 Contra Costa Regional Health Center, Martinez, CA, USA; 7 Department of Emergency Medicine, Yale University School of Medicine, New Haven, CT, USA

## Abstract

While primary care, obstetrical, and surgical services have started to expand in the world's poorest regions, there is only sparse literature on the essential support systems that are required to make these operations function. Diagnostic imaging is critical to effective rural healthcare delivery, yet it has been severely neglected by the academic, public, and private sectors. Currently, a large portion of the world's population lacks access to any form of diagnostic imaging. In this paper we argue that two primary imaging modalities--diagnostic ultrasound and X-Ray--are ideal for rural healthcare services and should be scaled-up in a rapid and standardized manner. Such machines, if designed for resource-poor settings, should a) be robust in harsh environmental conditions, b) function reliably in environments with unstable electricity, c) minimize radiation dangers to staff and patients, d) be operable by non-specialist providers, and e) produce high-quality images required for accurate diagnosis. Few manufacturers are producing ultrasound and X-Ray machines that meet the specifications needed for rural healthcare delivery in resource-poor regions. A coordinated effort is required to create demand sufficient for manufacturers to produce the desired machines and to ensure that the programs operating them are safe, effective, and financially feasible.

## Diagnostic Radiology: A Neglected Essential Service

Diagnostic radiology is a major growth industry in the healthcare sector worldwide, but most citizens in rural and impoverished areas currently lack access to any form of imaging. While 96% of emergency departments in the United States have CT scanners [1], large swaths of rural populations in resource-poor countries lack access to basic ultrasound and X-Ray. Unfortunately, very little solid data exist that provide an accurate picture of the current global neglect [2-4]. Even where diagnostic imaging is available, the machines are often unreliable; in some surveys nearly 70% of the X-Rays in developing country settings do not work [5-7]. Where our organization, Nyaya Health, operates a district hospital in rural western Nepal, for example, we are just now deploying the third of three functioning X-Ray machines for over one million people; we recently introduced the first ultrasound program in the same region [8]. In fact, our motivation for this paper was the absolute dearth of resources, companies, and implementation mechanisms as we deployed diagnostic imaging services.

The key diagnostic imaging modalities for primary care and emergency services in rural areas are X-Ray and ultrasound; together, they are able to meet over 90% of the imaging needs of the population [4]. When these modalities are not readily available, lengthy transportation for appropriate diagnostic studies can significantly delay treatment and result in greatly increased costs to an already marginalized patient population. In rural western Nepal, for example, many patients must travel over ten hours, and some over two days, to reach an X-Ray facility; transportation alone often costs over a month's income. Providing clinical care in the absence of these essential diagnostic technologies also bears the risk of inappropriate treatment and missed diagnoses that can significantly impact health outcomes. These barriers result in both under-diagnosis and delayed diagnosis, resulting in increased morbidity and mortality for conditions such as tuberculosis, pneumonia, fractures, and maternal complications. Tables 1 and 2 present several common conditions in resource-poor settings for which diagnostic imaging services are required.

**Table 1:** Core Conditions Utilizing Ultrasound in Resource-Poor Settings

Type	Condition	Intervention	Skill Level	Necessity
Abdominal	Cephalopelvic disproportion	Cesarean section	Advanced	Moderate
	Ectopic pregnancy	Surgical management	Advanced	Moderate
	Retained products of conception	Dilation and Currettage	Advanced	High
	Abruptio placentae	Medical and surgical management	Advanced	High
	Peripartum hemorrhage	Medical management	Basic	Moderate
	Cholecystitis	Medical and surgical management	Advanced	High
	Tuberculosis (intra-abdominal)	Medical management	Basic	High
	Hydronephrosis	Medical and surgical management	Basic	High
	Abdominal trauma	Medical and surgical management	Advanced	High
	Abdominal masses	Medical and surgical management	Basic	High
Chest	Pleural effusion	Thoracentesis	Advanced	High
	Pneumothorax	Chest tube	Advanced	Moderate
	Hemothorax	Thoracentesis	Advanced	High
Cardiovascular	Deep vein thrombosis	Anticoagulation	Basic	High
	Cardiac failure	Medical management	Basic	Moderate
	Cardiac valve disease	Medical and surgical management	Advanced	High
	Pericardial effusion	Medical management and pericardiocentesis	Advanced	High
Orthopedic	Spine, skull trauma	Surgical management	Advanced	Moderate
	Pediatric Osteomyelitis	Medical management	Basic	Moderate
	Rib, pelvis trauma	Surgical management	Advanced	Moderate
Neurological	Neonatal hemorrhage	Medical management	Advanced	High
	Neonatal infection	Medical management	Advanced	Moderate
Procedural	Intravenous Access	Procedural guidance	Basic	Moderate
	Abscess	Procedural guidance	Basic	Moderate
	Arthrocentesis	Procedural guidance	Basic	Moderate
	Paracentesis	Procedural guidance	Advanced	High
	Thoracentesis	Procedural guidance	Advanced	High
	Pericardiocentesis	Procedural guidance	Advanced	High
	Foreign Body	Procedural guidance	Basic	Moderate
	Lumbar Puncture	Procedural guidance	Basic	Moderate

*Skill level refers to the skills required both for diagnosis and for subsequent intervention of the generalist practitioner who might perform sonagraphy. We indicate the skill level as such because we assume in the resource-poor context that the generalist practitioner would be required to perform the ultrasound, interpret the result, and perform the indicated intervention. Necessity refers to the need for the imaging modality in diagnosis and management of the condition listed.

**Table 2:** Core Conditions Utilizing X-Ray in Resource-Poor Settings

Type	Condition	Intervention	Skill Level	Necessity
Chest	Pneumonia	Medical management	Basic	High
	Tuberculosis	Medical management	Basic	High
	Pneumothorax	Chest tube placement	Advanced	High
	Pleural effusion	Thoracentesis	Advanced	High
	Cardiac failure	Medical management	Advanced	Moderate
	Hemothorax	Thoracentesis	Advanced	High
	Chronic obstructive pulmonary disease	Medical management	Basic	Moderate
	Asthma	Medical management	Basic	Moderate
	Lung abscess	Medical management	Advanced	High
	Occupational lung diseases	Medical management	Basic	Moderate
Limb	Long bone fracture	Reduction and fixation	Advanced	High
	Small bone fracture	Reduction and fixation	Advanced	High
	Osteomyelitis	Medical and surgical management	Basic	Moderate
	Dietary deficiency diseases (scurvy, rickets)	Nutrient supplementation	Basic	Moderate

*Skill level refers to the skills required both for diagnosis and for subsequent intervention of the generalist practitioner who might perform radiography. We indicate the skill level as such because we assume in the resource-poor context that the generalist practitioner would be required to interpret the film and perform the indicated intervention. Necessity refers to the need for the imaging modality in diagnosis and management of the condition listed.

In this paper we will outline the necessary considerations for implementing diagnostic radiology services in resource-poor settings, discuss modality options for both X-ray and ultrasound, and argue that it is both necessary and feasible to rapidly scale up these technologies. Several pressing, competing health needs must be considered in thinking about the character, size, and scope of a global imaging program. As we face regularly in rural Nepal, endemic malnutrition, lack of access to clean water, and insecure housing all are large-scale crises that diagnostic radiology has no impact upon. Additionally, the effectiveness of diagnostics depends upon the availability of therapeutics. As such, to make the most effective use of scarce resources, the scale-up of diagnostic imaging must coincide with an expansion in operations research and in managerial structures capable of overseeing the long-term maintenance, quality assurance, and financing of imaging programs, in addition to similar capacity and infrastructure development of basic public health services.

## Developing Effective Technology Strategies

The technical requirements for diagnostic imaging in resource-poor rural areas are vastly different from those in urban tertiary-care centers. Machines designed for resource-poor settings should a) be robust in harsh environmental conditions, b) function reliably in environments with unstable electricity, c) minimize radiation dangers to staff and patients, d) be operable by non-specialists, and e) produce high-quality images required for accurate diagnosis.

Maintenance of diagnostic imaging machines in rural areas is critical to ensuring the long-term effectiveness of programs. Rural health care facilities are often far away from centers where maintenance services are available; as high as 50% of all X-Ray machines in resource-poor areas are currently non-functional [9]. To minimize the risk of malfunction and disuse, machines should be designed to function with simple maintenance and should be accompanied with straightforward troubleshooting manuals that can be reviewed by a non-technician. Electronic moving parts should be minimized, and any complex circuitry should be housed in rugged casing resistant to water and physical damage. The reality is, however, that visiting technicians will be required on occasion. As in the case of Nyaya Health, every effort should be made to utilize any outside technician visits to help develop local capacity (see case study, below). This is conceptually similar to the notion that visiting doctors are most effective when they combine any direct clinical services with teaching of other healthcare providers.

Reliable electricity generation is of paramount importance to rural healthcare delivery. Many rural clinics and hospitals do not have three-phase power transmission (a form of electricity, available in most tertiary care center locations, where three alternating currents are provided out of phase of each other, instead of the single current provided in single phase), and do not have large inverters or battery systems capable of delivering power beyond 5-15 kilowatts. Main ("grid") electrical supply is often unreliable and subject to wide voltage fluctuations. To effectively scale-up radiology programs, imaging equipment must be designed to operate in such environments. A simple standard, which based on our experiences we believe to be feasible, would be for systems to have the power requirements of a typical laptop. This would entail an approximately 100 watt electrical rating supplied by a 5-15 amp outlet supplied directly to a battery that has a life of several hours. The battery should be capable of being charged safely and effectively even with wide fluctuations in voltage.

It is imperative to protect both patients and providers from the dangerous risks of excess radiation exposure. In resource-poor settings where appropriate room design and architectural specifications may be more challenging, imaging systems should be designed to prioritize minimal radiation scattering. Guidelines for X-Ray machines are available, having undergone significant testing and review by several Word Health Organization-sponsored panels [10]. The radiation risks of ultrasound are non-existent, which is a significant advantage of this imaging modality.

We posit that generalist practitioners can and should be trained in diagnostic imaging. An enormous shortage of trained healthcare providers is one of the most significant barriers to effective global health delivery [11], and diagnostic imaging is no exception. Regardless of what imaging solutions are chosen, a major challenge is having trained providers on-site capable of making evidence-based decisions of when to use diagnostic imaging, how to interpret the images, and how to adjust treatment plans based on those interpretations. Since typically only 3-10 images per day might be expected in these settings [12], specialized staff would typically be under-utilized, difficult to retain, and not cost-effective. In this vein, task-shifting to mid-level providers [13] including radiology technicians and nurses offers an optimal utilization of limited resources. As we have been doing in rural Nepal, combining task shifting with teleradiology to gain remote consultation and quality assurance can further optimize these resources [14].

Finally, in spite of the modifications made to operate effectively in the rural environment, image quality must be sufficient for accurate diagnosis. This latter point is critical, particularly because there are a large number of less expensive machines available throughout the world but which produce compromised images and are of questionable safety. Ongoing quality assurance and operations research, described below, can ensure that programs are meeting image quality standards.

## Diagnostic Ultrasound

Ultrasound is a core imaging modality for point-of-care diagnostics for the generalist physician [15-20]. Obstetric ultrasound is essential to detect high-risk pregnancies and identify the cause of peripartum hemorrhage. Complications during pregnancy constitute some of the most common causes of maternal mortality worldwide [21] and pose critical barriers to achieving the UN maternal health millennial goals. Effective obstetric imaging can be achieved by generalist physicians and midwives [21], and it has been proposed that generalist ultrasound plays an important part in achieving the UN millennium goals on maternal and child health [22].

Additionally, the diagnosis of a broad spectrum of non-obstetric presentations can be assisted through ultrasound (Table 2). These include pericardial and pleural effusions, intra-abdominal hemmorhage, organomegaly, pediatric osteomyelitis, hydronephrosis, intra-abdominal tuberculosis, and cholelithiasis [22]. Trauma is common in rural areas, and rapid ultrasound may effectively screen for significant thoraco-abdominal trauma, including pneumothorax, cardiac tamponade, and abdominal organ injuries. These applications can oftentimes be effectively managed by generalist physicians who receive more advanced sonography training. Further research is necessary to explore the full extent of ultrasound's use in diagnostics in resource-poor areas. This is particularly true because the evidence is based in wealthy settings where the sonagrapher is a specialist with a cart-based ultrasound machine, as opposed to most resource-poor settings where generalists are using small, portable machines. It remains to be seen whether this strategy is able to achieve the same level of diagnostic accuracy as is found in wealthier settings.

Ultrasound may be particularly helpful for procedural guidance. Both central and peripheral intravenous access may be aided by ultrasound. Access to fluid filled spaces for diagnosis and therapy include ultrasound-guided arthrocentesis, paracentesis, thoracentesis, and pericardiocentesis. Ultrasound can also determine the presence and extent of an abscess pocket, and may be able to identify and guide the removal of foreign bodies.

For ultrasound, no individual design is necessarily superior, although recommendations do exist through WHO manuals [24]. Portable ultrasound is likely the most feasible in terms of transportation and maintenance. Most portable ultrasounds can be powered by a typical 5A or greater electric outlet. An approximately 3.5 MHz convex transabdominal transducer will be the most widely used and have the broadest public health impact. A high frequency linear probe will be most useful for procedural assistance. Cardiac and endocavity transducers have the potential for more advanced diagnostic applications.

A WHO Study Group has published guidelines available online for the training of physicians and other health workers in diagnostic ultrasound [24]. For comprehensive ultrasound use, the WHO recommends that physicians should undergo training over 3-6 months including 300-500 ultrasound examinations that are tailored to the local epidemiology. For non-physician providers, there is significant variation in what is feasible, desirable, or mandated by law. The WHO Study Group suggests 250 abdominal, 50 pelvic, 50 first trimester, 200 second/third trimester examinations to meet proficiency in those areas. While these may be ideal guidelines, more focused applications may not require as much training, and a degree of flexibility should be allowed to ensure that training does not interfere with treatment access. On-the-job training, of midwives by rotating physicians, for example, could ensure that midwives do not have to leave their postings where they are critically needed to obtain the necessary sonography credentials. That said, ultrasound is a highly operator-dependent imaging modality, and care must be taken to ensure adequate training and continuing medical education. Quality assurance, through additional radiologists readings and feedback is a central component to maintaining high level ultrasonagraphy. In our work with Nyaya Health, we have used a satellite internet connection to provide quality assurance feedback on ultrasound examinations performed (see case study, below).

## Diagnostic Radiography

Among other pressing global health needs, the clinical and public health need for diagnostic radiography services globally is clear. A large number of patients presenting for routine primary and emergency care suffer from pulmonary or orthopedic conditions for which radiography imaging is critical to diagnosis and treatment (Table 2) [11-13,25]. Effectively addressing tuberculosis, which is rising in prevalence in some regions and presenting in atypical manifestations secondary to the HIV pandemic, requires readily accessible diagnostic X-Ray to assess sputum-negative infections. Community- and hospital-acquired pneumonias ideally warrant accurate X-Ray diagnosis so that antibiotics can be appropriately prescribed. Orthopedic disease and trauma disproportionately causes severe morbidity among the rural poor; effective management necessitates timely X-Ray evaluation in many cases.

The global standard for X-Ray design has been established by several World Health Organization working groups, which devised the World Health Imaging System for Radiology (WHIS-RAD) [26,27]. Originally conceived in the 1970s as the Basic Radiological System and renamed in 1993 as the WHIS-RAD, it sets the design standards and programmatic strategies for rural resource-poor settings. The system has been described extensively elsewhere [10,28-40]. Briefly, the primary design characteristics include: 1) fixed tube column with tube and cassette holder at a fixed distance on a rotating tube arm, which guarantee minimal scatter radiation; 2) 11 kW battery system that can be charged even in extremely unstable electricity environments, charged by a 15 amp wall outlet at 110/220 V; 3) minimal moveable parts that minimize servicing needs and reduce the risk of malfunction; 4) high quality and safety largely independent of the operator; 5) owing to minimal scatter radiation risk, there are minimal additional site requirements; 6) x-ray tube ratings sufficient to reliably produce high-quality images.

Beyond the machine itself, solid image processing is a key component of a diagnostic radiography program. While procurement of consumables for image processing is less of a logistical bottleneck than is the maintenance of the machine itself, analogue processing remains less desirable owing to challenges in storing the films, disposing of waste, and performing distant quality assurance on the images. Several groups have been developing digital retrofits of the WHIS-RAD. The up-front costs of digital technologies are significant, but the long-term benefits are substantial. Except in cases where excellent maintenance and servicing can be assured, however, we recommend having analogue as a back-up. In Nyaya Health's case, we chose analogue to start out of concerns about the costs and reliability of the digital system, though our long-term vision is to go digital (see case study below and table 3).

**Table 3:** Cost of Deploying X-Ray in Rural Nepal

X-Ray Facility	Capital	Operating*
WHIS-RAD Machine Purchase	$30,000	--
X-Ray room construction	$5,000	--
Machine Transportation	$7,500	--
Installation	$1,500	--
Training costs	$500	--
WHIS-RAD Servicing	--	$300
X-Ray Tube replacement**	--	$350
X-Ray room maintenance	--	$200
Technician salary	--	$1,000
Electricity	--	$50
**Digital Processing**		
CR processor	$25,000	--
Desktop (server)	$400	--
Electricity	--	$75
**Analogue Processing**		
Dark room construction	$3,000	--
Chemical processor	$1,000	--
Storage space construction	$3,000	--
X-Ray developing materials	--	$4,000
**Summary**		
*Total costs, digital*	*$69,900*	*$1,975*
*Total costs, analogue*	*$51,500*	*$5,900*
*Number of years to reach cost equivalence*		*5*

Notes: All costs in US dollars. *Operating costs calculated on a yearly basis. **Tube replacement, since it occurs approximately every 15 years, is annualized by dividing the cost by 15

For machines meeting the WHIS-RAD specifications, several textbooks and training materials are available, but the quality of X-Ray services, as with nearly every other health intervention, is largely determined by broader interventions that improve the recruitment, professionalism, and retention of healthcare providers. With the WHIS-RAD machine, the need for a highly-educated radiation technologist is minimized by its ease of use. Local healthcare providers such as health assistants or community health workers can be trained in two-four weeks using standardized training materials [41].

## Operations and Performance Standards

A central component to effective radiology service delivery in resource-poor settings will be the development of robust data monitoring and evaluation programs. Coordinated feedback programs ensure continuing medical education for on-site staff and improved quality of care for patients. To facilitate such assessment and programmatic revision, programs can utilize electronic databases with simple, low-bandwidth uploading strategies to engage radiology experts in separate locations. In this manner, selective, but regular, review of images through telemedicine collaborations can be used to improve clinical quality and perform continuing medical education that would otherwise not be possible. Our experience in rural Nepal has thus far been quite positive for clinical care and staff medical education [14].

In such evaluation programs, clinical process indicators (e.g. numbers of diagnostic imaging tests performed, number of radiation safety checks, number of servicing visits) and outcomes measures (e.g. accuracy of the diagnoses, outcome of cases diagnosed with pneumonia, fracture, or other conditions) should be accounted for. Ultimately, the central aim of any monitoring and improvement program should be to impact public health outcomes measures, particularly in terms of death and disability averted, of conditions in which radiology can play a quantifiable role in reducing the time to effective treatment.

## Deploying Diagnostic Imaging Services in Rural Nepal: The Case of Nyaya Health

**Figure 1:**
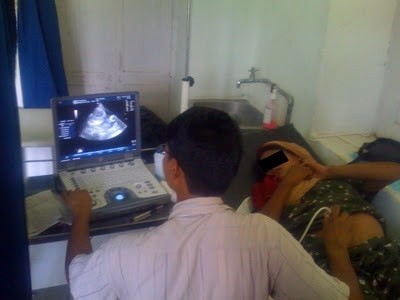
**Nyaya Health physician Dr. Jhapat Thapa performing ultrasound on a pregnant patient**.

Nyaya Health is a non-profit organization run by Nepal and US-based health professionals. In collaboration with the Nepali Ministry of Health & Population, Nyaya operates a hospital and regional health program in the district of Achham, one of the most remote and impoverished communities in South Asia. The district, just emerging from a decade-long civil war, has minimal health infrastructure; there were no allopathic physicians for a population of 250,000 people prior to our work there [42]. The roll-out of diagnostic imaging services proceeded step-wise, first with ultrasound services and more recently x-ray. In 2008, we started an ultrasound program using a GE LogicBook E machine provided by International Aid (Figure 1); this machine has an approximately US$40,000 value. We developed protocols for machine maintenance, appropriate use, and image transfer to the Yale Section of Emergency Medicine for review. The machine has been used by our physicians and mid-level providers for both obstetric and non-obstetric indications. Key challenges in the implementation of this program have included: a) lack of reliable electricity, b) technical problems with the machine without a nearby technician, c) frequent staff turnover following training leading to gaps in utilization, and d) damaged parts including electrical cords and plugs requiring sourcing from an international supplier. Image transfer has been interrupted by staff turnover and buy-in, and by electricity and telecommunications challenges. While the program has faced several challenges it continues to function today and remains an important part of our clinical services. All protocols are publicly available via the Nyaya Health wiki [14] and blog [8].

**Figure 2:**
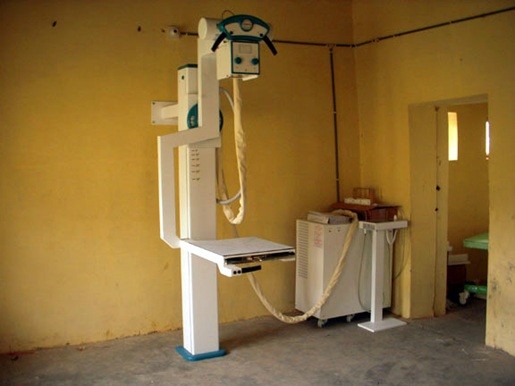
**Nyaya Health X-Ray Installation**. Note the protective screen around the control room had yet to be installed in this photograph.

Subsequently, in 2010, Nyaya Health initiated an X-Ray program with a WHIS-RAD machine from the Spanish company Sedecal (Figure 2). Prior to deployment, our team discussed and investigated options for manufacturers and models extensively, particularly whether to purchase from an in-country (Nepal) or international company. We consulted with numerous experts throughout the world, and in fact our frustration with the current state of knowledge about the scale-up of x-ray services was the primary motivation for this piece. Ultimately we selected the WHIS-RAD system for the reasons we have discussed here. Implementing the x-ray program proved to be significantly more challenging than ultrasound services. Our first challenge was delivering equipment that weighed over 750 kg to an extremely remote region with limited road access. This was further complicated by the need to have a technician onsite to install it; this required a team to travel from Dehli (India) over 2 days away. Onsite challenges included effective training of a local staff member and developing reliable electricity to charge and operate the WHIS-RAD system. We worked with a regional hospital to train a mid-level provider (4 weeks) and have agreed to a 2 year bonding period to ensure the staff member is not drawn to an urban area where work is available and living conditions are far better. The second major challenge onsite was the electricity situation. Even though the WHIS-RAD has a battery source, it requires a 110 or 220 V supply, and the main electricity grid where Nyaya works was only supplying a degraded 170 V signal; this problem was fixed with the addition of a relatively inexpensive voltage stabilizer yet delayed implementation by several months. Presently we are deploying analogue film processing, though we hope to scale to digital in the future. We have documented our work on our wiki [43] and blog [44]. In table 3, we provide the approximate costs of our x-ray deployment, which we used in planning services.

## Mobilizing Radiology Services for the Rural Poor

It is feasible to achieve significant gains in access to diagnostic imaging. A key strategy for achieving this is to develop a coordinated effort that can both leverage companies to produce machines meeting the specifications described here and convince donors and governments that the endeavor is worthwhile. A survey of diagnostic imaging services, coordinated by intergovernmental institutions such as the World Health Organization, could help guide the financing and logistics of global diagnostic imaging roll-out. Centralized financing, quality, and standards mechanisms such as have been achieved with the WHO's 3 × 5 initiative for HIV/AIDS or the Green Light Committee for tuberculosis provide successful models for how global radiology access could be achieved. By collectively mobilizing resources, academic institutions, governments, non-profit organizations, and private companies can together build a global network of diagnostic imaging services for the rural poor.

## Competing and Conflicting interests

All authors have completed the Unified Competing Interest form at http://www.icmje.org/coi_disclosure.pdf (available on request from the corresponding author) and declare that (1) DM, RS, SB, JA have no financial interests that may be relevant to the submitted work; (2) CM has contributed as a paid consultant for Philips Healthcare (ultrasound division) within the last two years. CM has contributed as a paid consultant for Sonosite, Inc. (ultrasound manufacturer) within the last year.

## Funding Sources

The authors report no funding sources for this article.

## Authors' contributions

DM conceived of the piece, drafted the initial manuscript, and read and approved the final piece RS, JA, SB, AS, and CM provided critical comments, edits, and literature reviews and read and approved the final piece.
